# Composition of Diagnostic Assessment Sheet Items for Developing a Personalized Forest Therapy Program for Patients with Depression: Application of the Delphi Technique

**DOI:** 10.3390/healthcare14010116

**Published:** 2026-01-03

**Authors:** Gayeon Kim, Sinae Kang, Kyungsook Paek, Youngeun Seo, Hyoju Choi, Seyeon Park, Pyeongsik Yeon

**Affiliations:** 1 Graduated Department of Forest Therapy, Chungbuk National University, Cheongju 28644, Republic of Korea; rkdus6520@naver.com (G.K.);; 2 Department of Forest Sciences, Chungbuk National University, Cheongju 28644, Republic of Korea

**Keywords:** depression, forest therapy, personalized intervention, Delphi technique, diagnostic assessment, healthcare, forest therapy activities

## Abstract

Background/Objectives: Depression is a serious mental illness worldwide, with a continuously increasing prevalence. As evidence supporting forest therapy as a non-pharmacological intervention has accumulated, the need for collaboration between the medical and forest therapy sectors has emerged; however, practical tools applicable in real-world clinical settings remain limited. This study aimed to derive components of a diagnostic assessment sheet to support clinicians in developing personalized forest therapy programs for patients with depression. Methods: Program-related literature and case materials from diverse disciplines were systematically analyzed to identify transferable program development elements and therapeutic activities. Based on these findings, a two-round Delphi survey was conducted with 17 experts in forest therapy and medicine. Results: Through the Delphi process, 26 therapeutic activities were identified and classified into six final activity types. Assessment items were developed to support clinicians in selecting appropriate activity types, and nine key precaution items were identified to enhance safety and appropriateness during program design and implementation. Conclusions: This study provides a structured framework to guide clinicians and forest therapy instructors in composing individualized forest therapy programs for patients with depression, supporting practical medical–forest therapy integration. Future research should validate the diagnostic assessment sheet through empirical field testing.

## 1. Introduction

Globally, the prevalence of depression, anxiety, and other mental disorders has been increasing, to the extent that these conditions are now perceived as common. Among mental disorders, depression constitutes a serious global public health concern, affecting about 3.8% of the world’s population—approximately 280 million people [1]. Depression is strongly associated with suicide; more than 700,000 people die by suicide each year, and South Korea has the highest suicide rate among OECD member countries [2]. If left untreated, mental disorders can lead to adverse outcomes; therefore, timely and accurate diagnosis and treatment are essential [3].

Without treatment, depression may persist for several months to over a year and lead to diverse difficulties in daily functioning [4]. Insomnia is among the most common symptoms, alongside gastrointestinal and respiratory symptoms and chronic fatigue [5]. Beyond the wide range of psychological and physical symptoms, approximately 85% of individuals with depression experience significant anxiety symptoms, and comorbid depression and anxiety are associated with longer treatment courses [6]. Feeling depressed at times is a natural part of life; however, if symptoms become severe enough to impair daily functioning, they warrant prompt evaluation and appropriate treatment [7]. In 2024, the National Center for Mental Health conducted a survey on mental health knowledge and attitudes to promote mental health and reported that, although understanding and acceptance of mental illness increased compared to 2022, the proportion of respondents who knew how to access mental health services decreased, underscoring the need for education on managing mental health problems and their importance [7]. Taken together, these findings highlight that depression is a condition requiring prompt and accurate treatment, reinforcing the importance of both effective therapeutic approaches and preventive strategies.

Treatment options for depression include pharmacological and non-pharmacological approaches. For pharmacological treatment, selection of an antidepressant is recommended based on the individual’s specific symptoms and relevant clinical factors [8]. Pharmacotherapy is the mainstay for severe depression; for mild to moderate depression, there is growing emphasis on combining pharmacological and non-pharmacological treatments [5]. In this context, interest in forest therapy as a complementary non-pharmacological modality has grown substantially, along with a corresponding rise in empirical research. Forest therapy is defined as “an activity that utilizes various elements of nature, such as scents and scenic views, to strengthen immunity and promote health” [9].

Forest therapy programs can be broadly categorized into guided programs, in which a forest therapy instructor conducts structured activities with participants, and self-guided programs, in which participants engage in activities independently. Guided programs have demonstrated sustained effectiveness, as participants experience the forest environment through structured facilitation by a trained instructor. However, the requirement of continuous instructor involvement may limit participants’ autonomy, leading to increased interest in self-guided alternatives. Ibes et al. (2018) [10] reported that university students who participated in a self-guided forest therapy program utilizing therapeutic signage along walking trails experienced reductions in stress and improvements in psychological well-being. Similarly, Kim (2021) [11] found that self-guided forest therapy produced positive effects comparable to guided programs in mood, stress responses, self-esteem, and happiness. More recently, Rivieccio et al. (2025) [12] demonstrated through a controlled comparison that therapist-guided forest immersion yields greater short-term mental-health benefits and higher economic value than self-guided immersion, highlighting the conditions under which professionally guided forest therapy may offer superior outcomes. Collectively, these findings support ongoing interest in both guided and self-guided approaches and underscore the broad therapeutic potential of forest engagement.

The therapeutic mechanisms underlying forest environments are understood to arise from multiple environmental factors, including clean air and water, high oxygen levels, phytoncides, thermal conditions, and biodiversity [13]. In particular, phytoncides—volatile organic compounds such as monoterpenes and sesquiterpenes—have been shown to enhance immune function [14] and improve sleep quality [15]. Additional studies have demonstrated that exposure to these compounds contributes to stress reduction [16]. Large-scale observational evidence further supports these findings; Donelli et al. (2023) [17] showed that exposure to plant-emitted monoterpenes was associated with reduced anxiety symptoms in a propensity-matched cohort, suggesting clinically meaningful psychophysiological effects. Recent work has also examined environmental variability, with Dudek et al. (2025) [18] reporting significant differences in terpene concentrations between younger and older forests, offering implications for optimizing forest sites used in therapeutic interventions. Thus, growing scientific evidence—including recent international clinical, environmental, and health-economics studies—indicates that forest environments positively influence human health in diverse ways.

Accumulated research further suggests that participation in forest therapy programs is associated with improvements in depressive symptoms [19] and reductions in depression and anxiety levels [20], supporting the depression-relieving effects of forest therapy. Accordingly, studies on forest therapy programs have expanded from the general population to patients with depressive disorders; forest activities have demonstrated effectiveness in reducing depressive symptoms in patients with depression [21], and urban-forest–based programs have likewise shown depressive symptom reductions [22,23]. Taken together, these findings support the effectiveness of forest therapy programs for patients with depression. Yeon et al. (2021) [24] characterized forest therapy activities as preventive management and a non-pharmacological treatment for improving depression and anxiety and recommended investigating optimal forest characteristics and structured activities to reduce depressive symptoms.

As evidence for forest therapy has accumulated, proposals have emerged to strengthen linkages with the medical sector. Zhang et al. (2022) [25] argued that forest therapy, by delivering multiple health benefits, may contribute to addressing public health challenges and called for stronger collaboration with the medical sector. The Republic of Korea’s Fifth and Sixth Forest Basic Plans emphasize activating linkages with the medical sector and report ongoing efforts to strengthen such collaboration, and Park (2025) [26], in an analysis of forest therapy research trends, likewise recommends further policy development and research to promote collaboration with the medical and pharmaceutical fields.

Internationally, nature-based prescriptions are already being implemented under various terms such as “Nature Prescription”, “Green Prescription”, and “Park Prescription”, whereby physicians or healthcare providers prescribe time or activities in natural environments [27]. Several countries—including the United Kingdom, the United States, Canada, and New Zealand—have integrated nature prescriptions into their healthcare systems. Notably, New Zealand’s Green Prescription (GRx) program enables physicians to prescribe community-based activities (e.g., mountain biking, hiking, walking) for patients with chronic diseases as part of a comprehensive health management service [28].

While these domestic and international cases demonstrate that healthcare providers can collaborate with forest-related services to deliver nature-based prescriptions or forest therapy activities, instances in which forest therapy instructors develop programs based explicitly on clinicians’ individualized clinical assessments remain exceedingly rare. For effective integration of forest therapy within healthcare settings, it is essential to establish tools and methods that enable forest therapy practitioners and medical professionals to fulfill their respective roles while maintaining direct, clinically meaningful linkages.

However, clinicians who would apply such programs in clinical settings often lack sufficient understanding of forest therapy, and it is difficult to propose programs to patients when the characteristics and clinical status of individuals with depression have not been adequately considered. Accordingly, as with prescribing medication, clinicians should directly assess patients’ characteristics and clinical status, identify suitable activities and key precautions for program delivery, and develop and provide tailored forest therapy programs.

In line with this need, the present study aims to develop a diagnostic assessment sheet to support the creation of tailored forest therapy programs for patients with depression. To achieve this, we conducted a literature review and Delphi analysis to identify forest therapy activities appropriate for individuals with depression, classify these activities into activity types, and derive key precautions that should be considered when providing programs. Ultimately, by developing a diagnostic assessment tool grounded in expert consensus, this study seeks to establish a systematic framework for medical–forest therapy linkage and enhance the clinical applicability of tailored forest therapy programs.

Specifically, the diagnostic assessment sheet proposed in this study is not intended as a diagnostic tool for depression but as a decision-support tool used after a clinical diagnosis has been established to evaluate the applicability of forest therapy and to guide program composition. Given the lack of standardized activity structures and precaution guidelines for forest therapy programs, this study systematically organizes applicable forest therapy activities and key precautions for patients with depression in the form of a diagnostic assessment sheet. The assessment sheet is designed for use by board-certified psychiatrists, who select appropriate activity types, specific activities, and precaution items based on comprehensive clinical judgment of individual patient characteristics and conditions. This structured clinical information is then communicated to certified forest therapy instructors, enabling the development of personalized forest therapy programs that integratively consider program goals, therapeutic stages, and intervention settings. Through this process, the present study provides a practical mechanism for bridging clinical assessment and forest therapy program design and offers a foundation for strengthening medical–forest therapy linkage.

## 2. Methods

### 2.1. Study Procedure

**Figure 1:**
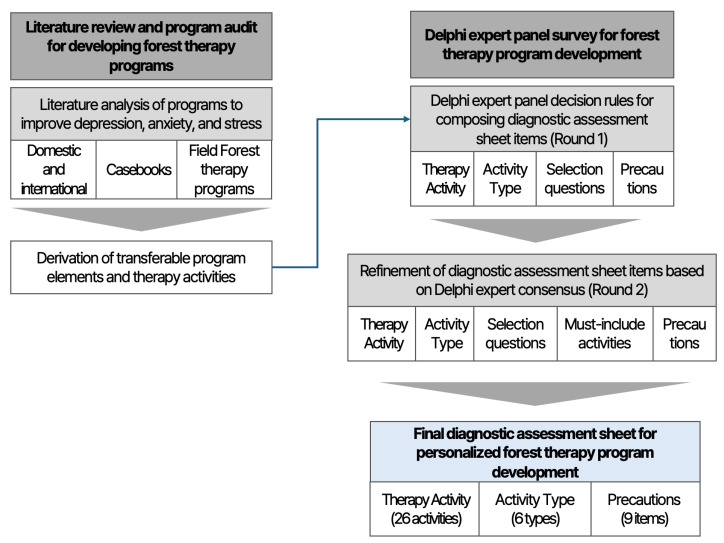
Conceptual workflow for developing a diagnostic assessment sheet for personalized forest therapy programs. This framework was developed based on prior research on forest therapy program development and expert consensus–based approaches, including literature and program analyses and Delphi survey methods [29,30].

This study conducted a literature-based program analysis and a Delphi survey to develop a diagnostic assessment sheet that supports the design of personalized forest therapy programs for patients with depression. The overall study procedure is conceptually illustrated in Figure 1.

To derive items for constructing the diagnostic assessment sheet, a comprehensive literature review and program analysis were first conducted on intervention programs aimed at improving depression-related symptoms, including depression, anxiety, and stress. In this process, the scope of analysis was not limited to forest therapy programs; rather, it encompassed various types of interventions, such as nature-based interventions, psychosocial activity programs, and other non-pharmacological interventions. This approach was adopted to identify common and transferable program components and activity characteristics that could be applied to the development of forest therapy programs.

Based on the findings from the literature review and program analysis, a Delphi survey questionnaire was developed. Through two rounds of the Delphi survey, forest therapy activities and activity types appropriate for patients with depression were identified. In addition, assessment items for selecting activity types based on individual characteristics and individual-level precaution items to be considered during forest therapy program delivery were derived. In the second round of the Delphi survey, experts additionally evaluated which activities should be included as mandatory components of forest therapy programs.

Between May and December 2024, existing intervention programs developed to improve depression-related symptoms were collected and analyzed to derive program components and therapeutic activities. Based on these findings, two rounds of the Delphi survey were conducted via email between 13 March and 17 April 2025. Through this process, this study aimed to construct diagnostic assessment sheet items that support clinicians in selecting appropriate forest therapy program components, activity types, and precaution items according to individual characteristics and conditions during the clinical assessment stage. This study was approved by the Institutional Review Board of Chungbuk National University (CBNU-2025-A-0020).

### 2.2. Delphi Survey Questionnaire

For Round 1, we developed questionnaire items based on program elements and therapy activities derived from the analysis of program-related literature and administered the first Delphi survey. The Round 2 questionnaire was then developed based on the Round 1 results. Through the Delphi survey, we gathered expert opinions on ‘selection and typology of forest therapy activities for patients with depression’ and on ‘evaluation items and precautions for selecting forest therapy activity types.’

#### 2.2.1. Literature Analysis

The literature analysis focused on program-related literature addressing improvements in depression, anxiety, and stress—key symptoms in patients with depression. To analyze programs developed to improve depression, anxiety, and stress in diverse fields in Korea and abroad, we collected and analyzed research papers, program casebooks, and on-site programs. The program fields were: forest therapy; healthcare/medicine/nursing; horticultural welfare/therapeutic farming; psychotherapy; social welfare; and exercise/rehabilitation.

Seven exclusion criteria were applied when selecting program data. Studies that did not include sufficiently detailed program plans were excluded because extracting specific therapy activities was essential for this analysis. Full-text screening was conducted to extract eligible programs using the following criteria: (1) full-text files were unavailable; (2) the program was not related to depression, anxiety, or stress; (3) the program was unrelated to program development; (4) the program analyzed the effectiveness of an already developed program rather than presenting original content; (5) the program consisted solely of educational activities rather than therapeutic activities; (6) detailed program plans were unavailable; and (7) the program required specialized qualifications for implementation.

**Figure 2:**
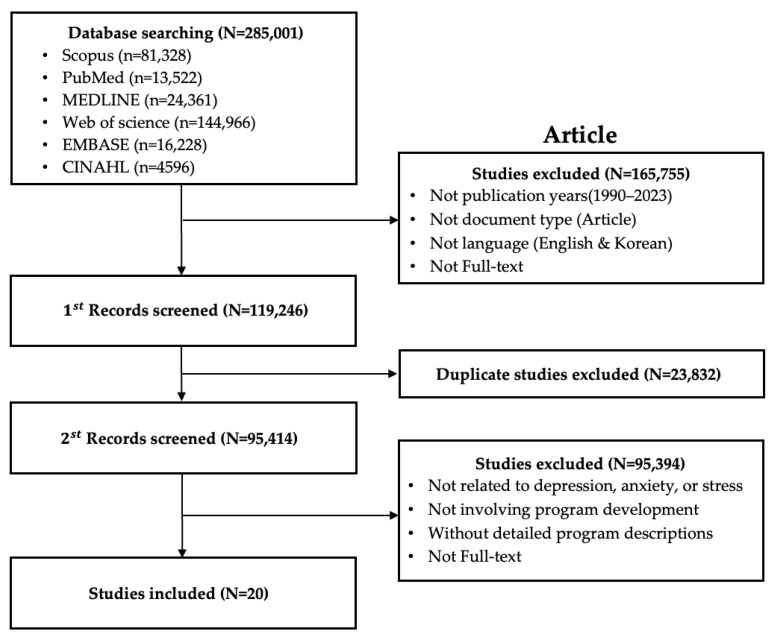
Flow chart (international articles).

**Figure 3:**
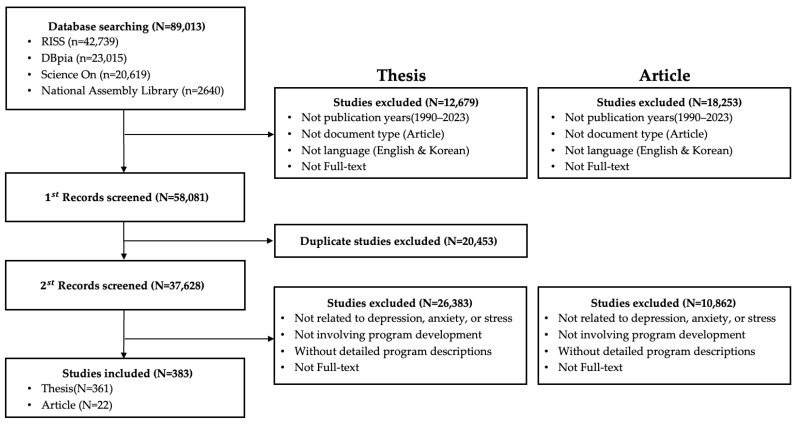
Flow chart (Korean articles).

We searched SCOPUS, PubMed, MEDLINE, Web of Science, EMBASE, CINAHL (Cumulative Index to Nursing and Allied Health), RISS (Research Information Sharing Service), DBpia, ScienceON, and the National Assembly Library for domestic and international journal articles and domestic theses published from 1990 to 2023, identifying 403 records through screening. The research literature selection process is presented in Figure 2 and Figure 3. In addition, we collected casebooks published by national institutions and, after screening, selected casebooks in three fields (forestry, healthcare/medicine, and therapeutic farming), from which 55 programs were identified. Regarding programs currently implemented in forest therapy settings, we surveyed seven organizations operating the ‘Mental Health (Stress) Recovery Support Program’ and 28 forest welfare companies, and collected nine programs conducted by three forest welfare institutions.

#### 2.2.2. Derivation of Therapy Activities for Forest Therapy Programs

We collected and analyzed programs across diverse fields to derive therapy activities applicable to forest therapy programs. Program data were extracted by six researchers (Gayeon Kim, Sinae Kang, Kyungsook Baek, Youngeun Seo, Hyojoo Choi, and Seyeon Park) according to predefined coding criteria. The research team cross-checked all extracted data and excluded individual subjective interpretations. Any discrepancies that arose during data extraction and analysis were resolved through discussion among the researchers. In classifying and integrating the extracted content, we sought to adhere as closely as possible to the categorization standards used within each field from which the literature originated.

The therapy activities derived from the programs were classified according to two criteria: (1) activities with an explicit purpose, developed accordingly (purpose-driven activities); and (2) activities not centered on a specific purpose and usable in various ways (practical activities). Nineteen purpose-driven activities and 40 practical activities were identified, for a total of 59 therapy activities (Table 1). The derived therapy activities were classified into eight activity types through six researcher meetings, and the therapy activities by activity type are shown in Table 2.

#### 2.2.3. Construction of the Round 1 Delphi Survey Questionnaire Items

Based on the therapy activities derived from the literature analysis and discussions among the research team, we constructed the items for the Round 1 Delphi survey. The Round 1 questionnaire gathered ratings of appropriateness and additional comments on: (1) selection and typology of forest therapy activities for patients with depression, and (2) evaluation items and precautions for selecting forest therapy activity types. For (1), the 59 therapy activities identified in the literature analysis were presented, classified into eight activity types. After presenting these, we asked respondents to indicate ‘type appropriateness’ using a binary O/X response and to rate ‘activity appropriateness’ on a 5-point Likert scale. For (2), we asked respondents to rate, on a 5-point Likert scale, the appropriateness of the questions intended to select forest therapy activity types. In addition, when developing and implementing forest therapy programs for patients, respondents were invited to freely provide opinions—via an open-ended question—on precautions that forest therapy instructors should consider. For all items, open-ended fields were provided for any additional comments. The main contents of the Round 1 Delphi questionnaire are summarized in Table 3.

#### 2.2.4. Construction of the Round 2 Delphi Survey Questionnaire

Based on the opinions collected in Round 1, we developed the Round 2 Delphi survey questionnaire. Using the Round 1 item means, content validity ratio (CVR) values, and additional comments, the research team convened to review each item; items that did not meet the CVR threshold were deleted or revised. For transparency, the item-wise means, CVR values, and details of deletions/revisions were explicitly presented in the Round 2 questionnaire so that experts could verify them. In Round 2, we added an assessment of the appropriateness of ‘activities that must be included when composing a forest therapy program’ to the existing Round 1 content. In addition, drawing on the open-ended responses about ‘precautions during program implementation’ from Round 1, we formulated items to assess the importance of each precaution. The main contents of the Round 2 Delphi questionnaire are summarized in Table 4.

### 2.3. Composition of the Expert Panel

For this study, we convened a panel of 20 experts, considering their credibility and level of engagement in relevant fields. Because no diagnostic assessment sheet for developing tailored forest therapy programs currently exists either domestically or internationally, the present study aimed to first develop a tool applicable within the Republic of Korea. Accordingly, the Delphi survey was conducted exclusively with Korean experts. Because the Delphi survey addressed forest therapy programs for patients with depression, we selected 10 psychiatrists and 10 experts in forest therapy as panel members. Eligibility required at least five years of experience in a relevant field or participation in related research or projects within the past five years. The composition of the expert panel participating in the Delphi survey is shown in Table 5.

### 2.4. Data Analysis

For the Delphi survey results, we used Microsoft Excel to calculate and present, for each item, the mean and the content validity ratio (CVR). All analyses were conducted using Microsoft Excel for Mac (version 16.101.3), and no additional statistical software packages were used. CVR values were computed based on Lawshe’s (1975) [31] content validity ratio. The CVR ranges from −1.0 to +1.0; a positive value indicates that at least half of respondents rated the item 4–5 on a 5-point Likert scale, a value of 0 indicates that exactly half did so, and a negative value indicates that at least half rated it 3 or below [32]. The minimum acceptable CVR depends on the panel size. According to Lawshe (1975) [31], when the number of respondents is around 20, the minimum CVR is 0.42. According to Ayre and Scally (2014) [33], when there are 17 respondents, the minimum CVR is 0.529, with a minimum of 13 essential endorsements required.

In our Round 1 Delphi survey, we initially intended to delete all items with a CVR below 0.529; however, there were discrepancies with experts’ additional comments in the questionnaire. After consultation among six researchers, we selected items for deletion by additionally considering the number of 1–2 ratings. When experts indicated in their open-ended responses that a given activity could be beneficial for patients, or when opinions between experts from different fields were not aligned, the item was retained for Round 2 to allow for re-evaluation, even if the CVR was as low as 0.41. In Round 2, all items with a CVR below 0.529 were deemed inappropriate.

## 3. Results

### 3.1. Round 1 Delphi Survey Results

The Round 1 Delphi survey for composing items of the diagnostic assessment sheet was administered to 20 experts; three dropped out, and 17 experts ultimately participated. Dropout occurred either because responses were not submitted within the designated period or because the experts themselves indicated that they did not consider their expertise to be sufficiently aligned with the topic and voluntarily withdrew from participation. Importantly, the dropout cases were not concentrated within a specific professional group but occurred across expert fields, and therefore did not lead to any imbalance in the panel composition or bias in the results. The results for (1) selection and typology of forest therapy activities for patients with depression and (2) evaluation items and precautions for selecting forest therapy activity types are as follows.

#### 3.1.1. Results of Round 1: Selection and Typology of Forest Therapy Activities for Patients with Depression

In the section on selection and typology of forest therapy activities for patients with depression, we gathered opinions on ‘type appropriateness’ and ‘activity appropriateness.’ First, for ‘type appropriateness’—whether the 59 forest therapy activities derived from the literature review were appropriately classified into eight activity types—we collected O (yes) and X (no) responses. If fewer than 13 experts responded O (yes), the activity was judged inappropriate for that type. Accordingly, among the 59 activities, those judged inappropriately classified by activity type were ‘mind–body training exercise’ under ‘sensory stimulation and mind–body relaxation activities’ (O = 11, X = 6) and ‘therapy equipment experience’ under ‘nature-based activities’ (O = 11, X = 6).

Next, for ‘activity appropriateness’—whether it is appropriate to offer the 59 forest therapy activities as components of a patient-tailored forest therapy program—12 items were deleted out of the 59: naming (assigning nicknames), mind–body training exercise, massage, recalling the past, expressing through movement, role-play, dancing, brain-stimulation exercise, video media activity, therapy equipment experience, insect-mediated activities, and animal-assisted activities. Although tea ceremony, awakening the five senses, setting my dreams and goals, yoga, music activities, crafting, cooking activities, storytelling, forest literature activities, indoor gardening, and assigning practice tasks showed CVR values of 0.41, only 0–2 experts rated them 1–2, and the reasons for negative responses were integration with other activities or mismatch with the designated activity type. Therefore, following a meeting of six researchers, these items were retained and included in the Round 2 Delphi survey. The item-wise means and CVR values for activity appropriateness are presented in Table 6, with deleted items shaded in gray.

#### 3.1.2. Results of Round 1: Evaluation Items and Precautions for Selecting Forest Therapy Activity Types

In the section on evaluation items and precautions for selecting forest therapy activity types, we collected opinions on the ‘appropriateness of selection questions for activity types’ and on ‘precautions when composing and implementing forest therapy programs for patients.’ The ‘selection questions for activity types’ are intended to determine whether a given activity should be offered to the patient when developing a forest therapy program and, accordingly, to help select the appropriate activity type; we examined whether the questions for each type were appropriate. Of the eight types, ‘introductory-stage activities’ were excluded from the selection questions on the premise that they must be included when composing a program. For the seven remaining types, all content validity ratio (CVR) values for the ‘appropriateness of selection questions for activity types’ were at or above 0.529, indicating that the diagnostic item content was appropriate. The item-wise means and CVR values for the appropriateness of selection questions are shown in Table 7.

In addition, experts were invited to freely provide open-ended comments on ‘precautions when composing and implementing forest therapy programs for patients.’ Their opinions are summarized in Table 8.

#### 3.1.3. Open-Ended Comments by Item from the Round 1 Delphi Survey

At the end of the closed-ended response sheet for Round 1, we provided open-ended fields for each item so that experts could freely add comments. The experts’ opinions are summarized in Table 9.

#### 3.1.4. Changes Based on Round 1 Delphi Survey Results

Based on the Round 1 results, we revised the therapy activities, activity types, and selection questions for activity types. The changes by item are shown in Table 10.

### 3.2. Results of the Round 2 Delphi Survey

The Round 2 Delphi survey for developing the diagnostic assessment sheet was conducted with the same 17 experts who participated in Round 1. The results for (1) selection and typology of forest therapy activities for patients with depression and (2) evaluation items and precautions for selecting forest therapy activity types are as follows.

#### 3.2.1. Results of Round 2: Selection and Typology of Forest Therapy Activities for Patients with Depression

In the section on selection and typology of forest therapy activities for patients with depression, we gathered experts’ opinions on ‘type appropriateness’, ‘activity appropriateness’, and ‘must-include activities’. For type appropriateness—whether the 45 forest therapy activities were appropriately classified into eight activity types—responses were collected in the form of O (yes) and X (no). In Round 2, all items for type appropriateness were judged appropriate.

For activity appropriateness—whether it is suitable to offer the 45 forest therapy activities as part of a patient-tailored forest therapy program—14 of the 45 items were deleted. The deleted items were: setting my dreams and goals, identifying strengths and weaknesses, cognitive restructuring and awareness, identifying and planning solutions, implementing and evaluating solutions, tea ceremony, play, literature activities, forest literature activities, music activities, laughter activities, cooking activities, storytelling, and assigning practice tasks. The item-wise means and CVR values for activity appropriateness are presented in Table 11, with deleted items shaded in gray.

In the Round 2 Delphi survey, an additional item—‘must-include activities’—was introduced to identify activities that should be considered essential components when composing a forest therapy program. Expert opinions were collected on whether ‘introductory-stage activities’ and ‘activity wrap-up and real-life linkage activities’ are appropriate as must-include activities. The results indicated that both items were judged to be appropriate as must-include activities (Table 12).

#### 3.2.2. Results of Round 2: Evaluation Items and Precautions for Selecting Forest Therapy Activity Types

In the section on evaluation items and precautions for selecting forest therapy activity types, expert opinions were obtained on the ‘appropriateness of selection questions for activity types’ and on ‘precautions when composing and implementing forest therapy programs for patients.’ For the appropriateness of selection questions, all CVR values for the seven activity types were at least 0.529, indicating that the content of the diagnostic items was appropriate. The item-wise means and CVR values for selection-question appropriateness are shown in Table 13.

Based on the Round 1 Delphi survey feedback, we developed items for ‘precautions when composing and implementing forest therapy programs for patients’ and examined the degree of importance of each precaution. The ‘importance’ ratings assessed whether each precaution was necessary to consider in program composition and implementation. The results showed that of the ten items, one item (The patient feels anxiety when engaging in activities that deeply explore their inner world) had a CVR value below 0.529 and was therefore deleted. The item-wise means and CVR values for the importance ratings of the precautions are presented in Table 14, with deleted items shaded in gray.

#### 3.2.3. Open-Ended Comments by Item from the Round 2 Delphi Survey

At the end of the closed-ended response sheet for Round 2, we provided open-ended fields for each item so that experts could freely offer additional comments. The experts’ opinions are summarized in Table 15.

#### 3.2.4. Changes Based on Round 2 Delphi Survey Results

Based on the results of the Round 2 Delphi survey, revisions and refinements were made to the forest therapy activities, activity types, selection questions for activity types, and precaution items. The specific changes made to each category are presented in Table 16.

The final activity types derived through the Delphi survey, along with the corresponding selection questions for each activity type and the detailed therapy activities, are presented in Table 17. The precaution items identified through expert consensus are presented in Table 18. Based on clinical judgment, a board-certified psychiatrist selects the activity types and specific therapy activities required for each patient and reviews and documents the precaution items that should be considered during participation in forest therapy programs. This information is then communicated to a certified forest therapy instructor, who develops a personalized forest therapy program by integratively considering the program goals, therapeutic stages, and the intervention setting (environment).

**Figure 4:**
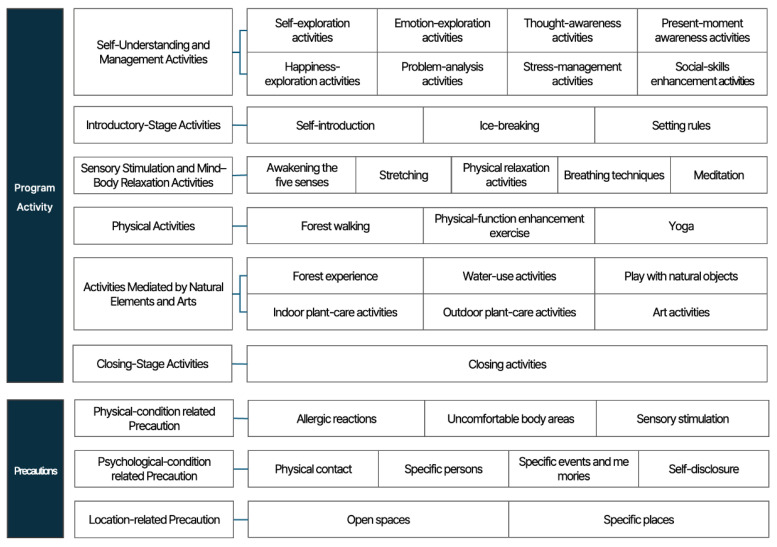
Summary of program activities and precautions for personalized forest therapy programs.

Table 17 and Table 18 are presented to explain the conceptual framework and key components of forest therapy program composition, and the activities and precaution items are summarized in Figure 4. In addition, the diagnostic assessment sheet designed for practical use in clinical and field settings is provided in Appendix A.

## 4. Discussion

In this study, a literature review and Delphi survey were conducted to compose the items of a diagnostic assessment sheet that can be used in developing tailored forest therapy programs for patients with depression. To this end, we analyzed program-related literature addressing improvements in depression, anxiety, and stress to derive therapy activities, which then informed the construction of the Round 1 Delphi survey questionnaire. Two rounds of Delphi surveys were administered to 17 participants comprising forest therapy experts and psychiatrists, thereby finalizing the items and questions for the diagnostic assessment sheet. Based on these results, the main points of discussion are as follows.

First, this study identified 26 therapy activities and six activity types for composing tailored forest therapy programs for patients with depression, based on a literature review and Delphi analysis. Compared to previous studies that classified forest therapy activities, this study offers a more clinically applicable framework. Earlier studies often classified activities according to functional characteristics without sufficiently reflecting the mental health characteristics or clinical risk factors of the target population. For example, Lee et al. (2011) [34] categorized activities based on natural material use (e.g., crafts), forest space use, physical activities, and mental activities, and further described activities by therapeutic modality, goal, setting, facilitator, and difficulty. Kim et al. (2019) [35] classified activities by target group, location, sensory modality, therapeutic approach, and physical movement. Jeon et al. (2022) [36] analyzed depression-improving programs by life cycle stage, participant characteristics (e.g., multi-age groups, clinical populations, COVID-19–related distress), program goals, activity types, six therapeutic modalities, sensory modalities, season, session length, and location. Although these prior studies presented various classification criteria, the six activity types proposed in this study differ in that they were developed with explicit consideration of the psychological and physical characteristics of patients with depression and their applicability in clinical settings. Their appropriateness was validated through expert consensus, thereby offering more practical criteria for program design.

Second, this study derived nine precautions that should be considered when applying forest therapy programs to patients with depression. While previous forest therapy and psychosocial intervention literature has mentioned general considerations, few studies have systematically organized such precautions. The structured precautions proposed here therefore play an important role in enhancing program safety and individualization. Notably, the item stating that “activities involving deep exploration of one’s inner self may induce anxiety” was excluded because its CVR did not meet the minimum threshold. Experts agreed that such content represents a fundamental principle in designing interventions for patients with depression rather than an individual-specific precaution. This aligns with prior findings showing that excessive self-disclosure or intensive emotional exploration in early intervention stages may heighten emotional burden and increase crisis risk [37]. Similarly, patients with depression are known to experience intensified negative rumination and heightened anxiety when directed toward deep internal focus, warranting careful consideration [38]. Therefore, the exclusion of this item confirms that it functions as a general therapeutic principle rather than a patient-specific precaution, and the nine precautions derived in this study can serve as practical safeguards in program design.

Third, the diagnostic assessment sheet developed in this study is not a tool for diagnosing depression but a clinician-guided tool intended to assist psychiatrists in selecting appropriate forest therapy activity types and prescribing tailored programs based on each patient’s clinical status and individual characteristics. The psychiatrist completes the assessment sheet using clinical judgments formed during the consultation, and because the necessary information is naturally obtained during the clinical encounter, completing the sheet does not impose an additional time burden. Through this tool, clinicians can more clearly determine which activity types are suitable for each patient, which activities should be avoided, and which components must be included when composing a program. Moreover, the diagnostic assessment sheet serves as a practical guide for clinicians who may have limited familiarity with forest therapy by clarifying the structure and characteristics of the activities. It also provides forest therapy instructors with clinically informed directions for program implementation, thereby enhancing the effectiveness of medical–forest therapy linkage. However, for forest therapy instructors to safely support patients with depression, additional training on mental health characteristics—such as emotional regulation difficulties and activity-related risk factors—is necessary. Such training will further strengthen the practical utility of the diagnostic assessment sheet and improve the safety and effectiveness of tailored forest therapy programs.

Fourth, the diagnostic assessment sheet developed in this study serves as a complementary rather than a substitute tool for existing clinical diagnostic and symptom evaluation instruments such as the PHQ-9, BDI, HAM-D, and GAD-7. While these clinical tools are essential for assessing symptom severity and changes over time, they are insufficient by themselves for constructing forest therapy programs. Relying solely on health information can be risky when designing forest therapy interventions, as various psychological and environmental characteristics—such as emotional sensitivity, responses to sensory stimulation, and the degree of fear or avoidance associated with particular activities—must be carefully considered. These characteristics directly influence whether specific activities are suitable for a patient and whether certain activities should necessarily be included or excluded. Therefore, the diagnostic assessment sheet proposed in this study can function as a supplementary tool that incorporates such considerations and enables more refined tailoring of forest therapy programs. By providing a structured mechanism that integrates clinical judgment with forest therapy intervention planning, the tool offers a foundation for strengthening the linkage between medical treatment and forest therapy.

The diagnostic assessment sheet developed in this study is intended to support the clinical judgment of board-certified psychiatrists and to facilitate the design and linkage of forest therapy programs tailored to individual patient characteristics and conditions. This assessment sheet is not intended for the diagnosis of depression; rather, it is used as a reference tool after a clinical diagnosis has already been established to evaluate the feasibility of applying forest therapy and to guide decisions regarding program composition.

During outpatient clinical care, after comprehensively assessing a patient’s condition, a psychiatrist may propose participation in a forest therapy program as a complementary intervention when it is deemed appropriate. The target population for program application consists of patients with mild depressive disorder. The subsequent process of program development and delivery follows a structured sequence: (1) a clinical interview between the patient and the psychiatrist; (2) provision of information and recommendation regarding participation in a forest therapy program; (3) completion of the diagnostic assessment sheet and its transfer to a certified forest therapy instructor; (4) development of a personalized forest therapy program based on the assessment sheet; and (5) delivery of the program.

After program implementation, follow-up interviews with the patient are conducted to assess program suitability and determine whether continuation is appropriate. If changes in the patient’s condition or adjustments to the program are required, the diagnostic assessment sheet is completed again, and the program is revised or restructured accordingly. This process is designed to operate as an iterative feedback system rather than a one-time intervention, continuously reflecting the patient’s condition and responses.

The items derived from this study can serve as indicators of what should be prioritized when developing and conducting forest therapy programs for patients with depression. They are expected to aid in creating programs tailored to each individual. This finding is consistent with previous research asserting that customized forest therapy programs should be provided through a clear analysis of the target population during the program planning stage [34,36,39]. By presenting the diagnostic assessment sheet based on the derived items, this study is expected to contribute to the activation and broader adoption of tailored forest therapy programs.

## 5. Conclusions

This study conducted a literature review and Delphi survey to compose the items of a diagnostic assessment sheet for developing tailored forest therapy programs for patients with depression. This study identified forest therapy activity types, detailed therapy activities, and individual-specific precautions appropriate for patients with depression and organized corresponding selection questions for clinicians’ use, thereby laying a foundation for the development of tailored forest therapy programs.

This has important significance in that it allows programs to be composed systematically based on scientific evidence, departing from conventional program development approaches. Furthermore, by reflecting individual-specific rather than generic precautions, a more precise and safer approach to program development and implementation can be achieved.

Through this study, it is anticipated that even clinicians for whom forest therapy may be unfamiliar will be provided with a way to understand and approach forest therapy more easily, thereby systematizing linkage with the medical sector and enhancing its applicability in clinical settings. Ultimately, this study presents practical direction for developing individualized forest therapy programs for patients with depression and is expected to be utilized as an effective therapeutic intervention.

While the literature review and Delphi survey were conducted to derive results for composing items of a diagnostic assessment sheet for developing tailored forest therapy programs for patients with depression, this study has certain limitations.

First, although the intention was to propose therapy activities according to patients’ symptoms in order to develop the diagnostic assessment sheet, it was determined that there is currently no sufficient scientific evidence specifying which activities are effective for which symptoms. Therefore, we could not present therapy activities matched to specific symptoms. Continued detailed and scientifically rigorous research is required to establish clear evidence of which activities are effective for which symptoms.

Second, although activity types and therapy activities were presented to assist in program development, the therapy activities that can be provided may vary depending on the program’s objectives, the conditions of the forest environment, and the capacity of the forest therapy instructor. It is considered necessary to prepare a baseline set of guidelines to help reduce quality differences between programs.

Third, although the diagnostic assessment sheet items were derived through the Delphi survey, after developing a diagnostic assessment sheet reflecting these items, further elaboration is needed regarding its applicability in the field and instructions for its use. Empirical research should be conducted to secure validity in this regard.

Fourth, the Delphi survey was conducted exclusively with Korean experts. Because this limited the inclusion of international perspectives, future studies should incorporate experts from diverse countries and cultural contexts to enhance the global applicability and generalizability of the diagnostic assessment sheet.

Fifth, although the Delphi survey enabled the extraction of items for composing the diagnostic assessment sheet, additional quantitative validation procedures—such as exploratory or confirmatory factor analysis—were not conducted. Delphi results alone cannot fully establish the validity and reliability of the underlying constructs. Therefore, future research should conduct factor-analytic validation and reliability testing using larger samples to strengthen the structural validity of the diagnostic assessment sheet.

## Figures and Tables

**Table 1:** Therapy activities list.

	Therapy Activities		
Purpose-driven activities	Discovering my values	Analyzing the problem	Noticing stress
	Awakening the five senses	Identifying and planning solutions	Expressing stress
	Noticing emotions	Implementing and evaluating solutions	Cognitive restructuring and awareness
	Expressing emotions	Enhancing social skills	Identifying strengths and weaknesses
	Recalling the past	Setting my dreams and goals	Noticing the present
	Noticing thoughts	Finding ways to cope and manage stress	Finding happiness
	Self-exploration activities		
Practical activities	Assigning tasks	Ice-breaking	Brain stimulation exercise
	Setting rules	Role play	Forest experiences
	Play	Yoga	Insect-mediated activities
	Tea ceremony	Cooking activities	Animal-assisted activities
	Expressing through movement	Laughter activities	Creative activities
	Massage	Music activities	Hydrotherapy activities
	Crafting	Self-introduction	Forest walking
	Meditation	Playing with natural objects	Forest Literature activities
	Literature activities	Dancing	Forest workout
	Art activities	Breathing Techniques	Storytelling
	Naming (assigning nicknames)	Wrapping up an activity	Video media activity
	Stretching	Indoor Gardening	Exercise
	Physical relaxation	Outdoor Gardening	Therapy equipment experience
	Mind-body training exercise		

This table presents the activity classification used in the Round 1 Delphi survey; some items were revised or modified during subsequent Delphi rounds.

**Table 2:** Therapy activities typology.

Activity Type	Therapy Activities		
Introductory-stage activities	Ice-breaking	Self-introduction	Naming (assigning nicknames)
	Setting rules		
Sensory stimulation and mind-body relaxation activities	Meditation	Breathing Techniques	Physical relaxation
	Mind-body training exercise	Stretching	Massage
	Tea ceremony	Awakening the five senses	
Self-understanding and problem-solving activities	Discovering my values	Setting my dreams and goals	Noticing stress
	Noticing emotions	Noticing thoughts	Expressing stress
	Expressing emotions	Noticing the present	Analyzing the problem
	Recalling the past	Finding happiness	Identifying and planning solutions
	Self-exploration activities	Cognitive restructuring and awareness	Implementing and evaluating solutions
	Identifying strengths and weaknesses	Finding ways to cope and manage stress	
Social skills and relationship-building activities	Enhancing social skills	Role play	Play
	Expressing through movement		
Physical activity and exercise	Exercise	Dancing	Yoga
	Brain stimulation exercise	Forest workout	
Arts and creative activities	Art activities	Crafting	Literature activities
	Cooking activities	Video media activity	Creative activities
	Laughter activities	Music activities	Storytelling
Nature-based activities	Forest experiences	Indoor Gardening	Hydrotherapy activities
	Forest walking	Outdoor Gardening	Playing with natural objects
	Forest literature activities	Animal-assisted activities	Insect-mediated activities
	Therapy equipment experience		
Activity wrap-up and real-life linkage activities	Assigning tasks	Wrapping up an activity	

This table presents the activity classification used in the Round 1 Delphi survey; some items were revised or modified during subsequent Delphi rounds.

**Table 3:** Composition of the Round 1 Delphi survey questionnaire.

Survey Contents
(1) Selection and typology of forest therapy activities for patients with depression ① Type appropriateness (O/X response) - Were the 59 therapy activities appropriately classified into the 8 activity types? ② Activity appropriateness (5-point Likert scale) - Is it appropriate to offer the 59 therapy activities as activities in a patient-tailored forest therapy program? (2) Evaluation items and precautions for selecting forest therapy activity types ① Appropriateness of the selection questions for activity types (5-point Likert scale) - Are the content and wording of the diagnostic assessment items for selecting forest therapy activity types appropriate? ② Precautions when designing and delivering forest therapy programs for patients (open-ended question) - What psychological, physical, social, and environmental factors should be considered?

**Table 4:** Composition of the Round 2 Delphi survey questionnaire.

Survey Contents
(1) Selection and typology of forest therapy activities for patients with depression ① Type appropriateness (O/X response) - Were the 45 therapy activities appropriately classified into the 8 activity types? ② Activity appropriateness (5-point Likert scale) - Is it appropriate to offer the 45 therapy activities as part of a patient-tailored forest therapy program? ③ Must-include activities (5-point Likert scale) - When composing the program, is it appropriate to include ‘introductory-stage activities’ and ‘activity wrap-up and real-life linkage activities’ as mandatory components? (2) Evaluation items and precautions for selecting forest therapy activity types ① Appropriateness of the selection questions for activity types (5-point Likert scale) - Are the content and wording of the diagnostic assessment items for selecting forest therapy activity types appropriate? ② Importance of precautions for composing and implementing forest therapy programs for patients (5-point Likert scale) - Are these necessary items as precautions to consider when composing and implementing forest therapy programs?

**Table 5:** Expert panel list.

No.	Field	Gender	Affiliation & Position	Experience (Years)
1	Forest therapy	Female	Director, Research Institute under the Korea Forest Service	26
2		Female	Senior Researcher, Research Institute under the Korea Forest Service	18
3		Male	Head, Public Institution under the Korea Forest Service	16
4		Female	Team Leader, Public Institution under the Korea Forest Service	9
5		Female	Team Leader, Public Institution under the Korea Forest Service	13
6		Male	Director, National Healing Forest under the Korea Forest Service	15
7		Female	Ph.D., National Recreational Forest under the Korea Forest Service	13
8		Male	Professor, Department of Forest Environmental Resources, University	21
9		Female	Associate Professor, Department of Forest Science, University	21
10		Male	Assistant Professor, Department of Environmental & Forest Sciences, University	15
11	Psychiatry	Male	Director, Department of Psychiatry, D Hospital	9
12		Male	Director, Department of Psychiatry, S Hospital	8
13		Male	Director, Department of Psychiatry, O Hospital	6
14		Male	Director, Department of Psychiatry, Y Hospital	10
15		Male	Director, Department of Psychiatry, G Hospital	3
16		Male	Director, Department of Psychiatry, M Hospital	25
17		Male	Director, Department of Psychiatry, H Hospital	12
18		Male	Director, Department of Psychiatry, H Hospital	8
19		Male	Director, Department of Psychiatry, S Hospital	8
20		Male	Director, Department of Psychiatry, M Hospital	12

**Table 6:** Round 1 Delphi survey results (activity appropriateness).

Type	Sub-Activity	Mean	CVR	Type	Sub-Activity	Mean	CVR
Introductory-stage activities	Ice-breaking	4.29	0.65	Social skills and relationship-building activities	Enhancing social skills	4.31	0.65
	Self-introduction	4.35	0.53		Expressing through movement	3.76	0.18
	Naming(assigning nicknames)	4.00	0.29		Role play	3.71	0.06
	Setting rules	4.59	0.76		Play	4.12	0.65
Sensory stimulation and mind-body relaxation activities	Meditation	4.76	1.00	Physical activity and exercise	Exercise	4.24	0.65
	Breathing Techniques	4.88	1.00		Dancing	3.82	0.29
	Physical relaxation	4.71	1.00		Yoga	4.00	0.41
	Mind-body training exercise	3.82	0.29		Brain stimulation exercise	3.59	0.18
	Stretching	4.59	0.88		Forest workout	4.35	0.53
	Massage	3.65	0.06	Arts and creative activities	Art activities	4.06	0.53
	Tea ceremony	3.82	0.41		Crafting	4.00	0.53
	Awakening the five senses	4.12	0.41		Literature activities	4.00	0.53
Self-understanding and problem-solving activities	Discovering my values	4.29	0.76		Video media activity	3.71	0.18
	Noticing emotions	4.47	0.76		Music activities	3.88	0.41
	Expressing emotions	4.29	0.76		Laughter activities	4.00	0.53
	Recalling the past	3.82	0.29		Creative activities	3.82	0.41
	Setting my dreams and goals	4.00	0.41		Cooking activities	3.94	0.41
	Self-exploration activities	4.24	0.76		Storytelling	3.88	0.41
	Noticing thoughts	4.35	0.65	Nature-based activities	Forest experiences	4.65	0.76
	Noticing the present	4.24	0.65		Forest walking	4.76	0.88
	Finding happiness	4.24	0.65		Forest literature activities	3.76	0.41
	Identifying strengths and weaknesses	4.06	0.53		Indoor Gardening	3.82	0.41
	Noticing stress	4.18	0.88		Outdoor Gardening	4.00	0.65
	Expressing stress	4.06	0.65		Therapy equipment experience	3.47	0.06
	Finding ways to cope and manage stress	4.41	0.88		Hydrotherapy activities	4.18	0.53
	Cognitive restructuring and awareness	4.41	0.76		Playing with natural objects	4.12	0.65
	Expressing stress	4.18	0.65		Insect-mediated activities	3.47	0.18
	Identifying and planning solutions	4.29	0.65		Animal-assisted activities	3.59	0.06
	Implementing and evaluating solutions	4.29	0.65	Activity wrap-up and real-life linkage activities	Wrapping up an activity	4.59	0.76
					Assigning tasks	4.24	0.41

**Table 7:** Round 1 Delphi survey results (appropriateness of selection questions for activity types).

Activity Type	Diagnostic Question	Mean	CVR
Sensory stimulation and mind–body relaxation activities	Is there a need for activities that focus the patient’s attention on the senses and stabilize the body and mind?	4.35	0.76
Self-understanding and problem-solving activities	Is there a need for activities that help the patient develop an integrated self-understanding and problem-solving skills?	4.53	0.88
Social skills and relationship-building activities	Is there a need for activities that enhance social skills for forming and improving relationships with others?	4.29	0.76
Physical activity and exercise	Is there a need for activities that enable the patient to feel physical vitality?	4.59	1.00
Arts and creative activities	Is there a need for arts and creative activities that allow the patient to express themselves freely?	4.12	0.65
Nature-based activities	Is there a need for activities that explore and utilize elements of nature?	4.18	0.65
Activity wrap-up and real-life linkage activities	Is there a need for activities that support practice in daily life to sustain program effects?	4.29	0.65

**Table 8:** Round 1 Delphi survey open-ended responses (precautions).

Expert Opinions on Precautions for Composing and Implementing Programs
- Consider whether the patient may have allergic reactions that could occur during forest activities. - Consider whether the patient has uncomfortable body areas or medical conditions that warrant caution with specific activities. - Consider whether caution is needed regarding specific sensory stimuli. - Consider whether the patient is uncomfortable with physical contact with others. - Consider whether the patient has fears related to specific genders or age groups. - Consider whether the patient fears cues that may evoke particular events or memories. - Consider whether the patient is uncomfortable with activities that probe deeply into their inner experiences. - Consider whether the patient is burdened by expressing thoughts and feelings in front of others. - Consider whether the patient is uncomfortable engaging in activities in open spaces where they feel observed by others. - Consider whether the patient experiences anxiety in specific places such as near bodies of water or in dark spaces.

**Table 9:** **Round 1** Open-ended comments by item.

Item	Expert Comments
**Type appropriateness**	- The term ‘nature-based activities’ might be better expressed as ‘activities mediated by natural elements’ or ‘activities for communion with and experiencing natural elements.’ - There is a need to establish play activities as a separate activity type. - Wrap-up activities, like introductory-stage activities, should also be considered essential components. - Forest walking is more suitable for physical activity and exercise than for “nature-based activities”. - “Forest literature activities” are more suitable for “arts and creative activities”.
**Activity appropriateness**	- Sub-activities under self-understanding and problem-solving activities should be integrated where they are similar. - The distinctions among art activities, crafts, and making are unclear - The expressions “indoor gardening” and “outdoor gardening” are not intuitive.
**Appropriateness of selection questions for activity types**	- (Sensory stimulation and mind–body relaxation activities) focusing the patient’s attention on the senses would be better expressed as stimulating the patient’s senses. - (Sensory stimulation and mind–body relaxation activities) stabilizing the body and mind would be better expressed as relaxing the body and mind.

**Table 10:** Changes reflecting the Round 1 Delphi survey results.

Item	Changes
Activity types	- (Activity type) Deleted ‘social skills and relationship-building activities.’ - (Activity type) Added ‘play activities.’ - (Activity type) Renamed ‘nature-based activities’ to ‘activities mediated by natural elements.’ - (Reclassification of sub-activities) ‘Enhancing social skills’ reclassified under ‘self-understanding and problem-solving activities.’ - (Reclassification of sub-activities) ‘Forest walking’ reclassified under ‘physical activity and exercise.’ - (Reclassification of sub-activities) ‘Forest literature activities’ reclassified under ‘arts and creative activities.’ - (Reclassification of sub-activities) ‘Playing with natural objects’ reclassified under ‘play activities.’
Sub-activities	- Integrated ‘crafts’, ‘making’, and ‘art activities’ into ’art activities.’ - Renamed ‘indoor gardening’ to ‘indoor plant-use activities.’ - Renamed ‘outdoor gardening’ to ‘outdoor plant-use activities.’ - Deleted ‘naming (assigning nicknames)’, ‘mind–body training exercise’, ‘massage’, ‘recalling the past’, ‘expressing through movement’, ‘role-play’, ‘dancing’, ‘brain-stimulation exercise’, ‘video media activity’, ‘therapy equipment experience’, ‘insect-mediated activities’, and ‘animal-assisted activities.’
Selection questions for activity types	- (Self-understanding and problem-solving activities) Changed ‘for the patient to understand themselves’ to ‘for the patient to understand themselves and others.’ - (Sensory stimulation and mind–body relaxation activities) Changed ‘activities that focus the patient’s attention on the senses and relax the body and mind’ to ‘activities that stimulate the patient’s senses and relax the body and mind.’ - (Play activities) Added the question “Is there a need for activities that allow the patient to relieve tension and feel enjoyment in an active manner?” due to the addition of this activity type.

**Table 11:** Round 2 Delphi survey results (activity appropriateness).

Type	Sub-Activity	Mean	CVR	Type	Sub-Activity	Mean	CVR
Self-understanding and problem-solving activities	*Discovering my values*	4.24	0.76	Play activities	Play	3.94	0.41
	*Noticing emotions*	4.65	0.88		*Play with natural objects*	4.12	0.65
	*Expressing emotions*	4.24	0.65	Physical activity and exercise	*Exercise*	4.29	0.53
	Setting my dreams and goals	3.88	0.29		*Yoga*	4.00	0.53
	*Self-exploration activities*	4.29	0.65		*Forest exercise*	4.59	0.88
	*Noticing thoughts*	4.53	0.88		*Forest walking*	4.76	0.88
	*Noticing the present*	4.18	0.65	Arts and creative activities	*Art activities*	4.35	0.65
	*Finding happiness*	3.94	0.53		Literature activities	4.12	0.29
	Identifying strengths and weaknesses	3.76	0.29		Forest literature activities	3.59	−0.06
	*Noticing stress*	4.59	1.00		Music activities	4.12	0.41
	*Expressing stress*	4.24	0.65		Laughter activities	3.82	0.06
	*Finding ways to cope and manage stress*	4.18	0.65		Cooking activities	4.06	0.29
	Cognitive restructuring and awareness	3.94	0.18		Storytelling	4.12	0.41
	*Problem analysis*	4.06	0.53	Activities mediated by natural elements	*Forest experience*	4.53	0.76
	Identifying and planning solutions	4.12	0.29		*Indoor plant-use activities*	4.00	0.53
	Implementing and evaluating solutions	3.94	0.18		*Outdoor plant-use activities*	4.47	0.76
	*Enhancing social skills*	4.12	0.53		*Hydrotherapy activities*	4.29	0.65
Introductory-stage activities	*Ice-breaking*	4.35	0.76	Activity wrap-up and real-life linkage activities	*Concluding activities*	4.65	0.88
	*Self-introduction*	4.47	0.88		Assigning practice tasks	4.00	0.41
	*Setting rules*	4.35	0.76				
Sensory stimulation and mind-body relaxation activities	*Meditation*	4.65	0.88				
	*Breathing Techniques*	4.47	0.88				
	*Physical relaxation*	4.76	1.00				
	*Stretching*	4.59	1.00				
	Tea ceremony	4.00	0.41				
	*Awakening the five senses*	4.41	0.76				

**Table 12:** Round 2 Delphi survey results (must-include activities).

Activity Type	Mean	CVR
*Introductory-stage activities*	3.94	0.41
*Activity wrap-up and real-life linkage activities*	4.12	0.65

**Table 13:** Round 2 Delphi survey results (appropriateness of selection questions for activity types).

Activity Type	Diagnostic Question	Mean	CVR
Sensory stimulation and mind–body relaxation activities	*Is there a need for activities that focus the patient’s attention on the senses and stabilize the body and mind?*	4.65	1.00
Self-understanding and problem-solving activities	*Is there a need for activities that help the patient understand themselves and others and develop problem* *-* *solving skills?*	4.18	0.65
Play activities	*Is there a need for activities that allow the patient to actively release tension and experience enjoyment?*	4.29	0.77
Physical activity and exercise	*Is there a need for activities that enable the patient to feel physical vitality?*	4.41	0.88
Arts and creative activities	*Is there a need for arts and creative activities that allow the patient to express themselves freely?*	4.29	0.77
Activities mediated by natural elements	*Is there a need for activities that explore and use elements of nature?*	4.41	0.65
Activity wrap-up and real-life linkage activities	*Is there a need for activities that help patients practice in daily life to sustain the program’s benefits?*	4.35	0.77

**Table 14:** Round 2 Delphi survey results (precautions).

No.	Precaution Content	Mean	CVR
1	*The patient may be at risk of allergic reactions during forest activities. (Type of allergy: ________)*	4.65	0.88
2	*The patient has uncomfortable body areas or medical conditions that make certain activities impossible. (Specify: ________)*	4.76	1.00
3	*The patient feels discomfort when sensory stimulation becomes excessive.*	4.18	0.65
4	*The patient feels uncomfortable with physical contact with others.*	4.47	0.88
5	*The patient has fears related to specific persons (e.g., gender, age). (Characteristics of the person: ________)*	4.12	0.65
6	*The patient fears cues that may evoke particular events or memories. (Matters to be avoided: ________)*	4.24	0.65
7	The patient feels anxiety when engaging in activities that deeply explore their inner self.	3.88	0.41
8	*The patient feels burdened expressing thoughts and emotions in front of others.*	4.25	0.65
9	*The patient feels uncomfortable engaging in activities in open spaces where they feel observed by others.*	4.00	0.65
10	*The patient feels anxiety in specific places (e.g., near bodies of water, dark spaces, enclosed spaces). (Specify: ________)*	4.35	0.88

**Table 15:** **Round 2** Open-ended comments by item.

Item	Expert Comments
Type appropriateness	- It may be more appropriate for ‘play activities’ to belong within another activity type rather than as a separate type.
Activity appropriateness	- Sub-activities under ‘self-understanding and problem-solving activities’ should be integrated where they are similar. - The distinction between ‘forest exercise’ and ‘exercise’ is unclear; they should be integrated. - The term ‘exercise’ is too broad and requires renaming. - ‘Indoor plant-use activities’ and ‘outdoor plant-use activities’ could be renamed to ‘indoor plant-care activities’ and ‘outdoor plant-care activities’, as they focus on maintenance.
Appropriateness of selection questions for activity types	- For ‘self-understanding and problem-solving activities’, ‘sensory stimulation and mind–body relaxation activities’, and ‘physical activity and exercise’, the content is written mainly from a purpose perspective, which makes the concepts somewhat ambiguous. - The distinction between ‘play activities’ and ‘sensory stimulation and mind–body relaxation activities’ is unclear.
Importance of precautions	- The meaning of ‘excessive sensory stimulation’ is unclear. - ‘Activities that deeply explore the inner self’ should not necessarily be considered a patient-specific precaution but rather a general consideration for overall program implementation, so it may not need to be asked separately.

**Table 16:** Changes reflecting the Round 2 Delphi survey results.

Item	Changes
Activity types	- (Activity type) Deleted ‘Play activities’. - (Activity type) Deleted ‘Arts and creative activities’. - (Activity type) Renamed ‘Activities mediated by natural elements’ to ‘Activities mediated by natural elements and arts.’ - (Activity type) Renamed ‘Self-understanding and problem-solving activities’ to ‘Self-understanding and management activities.’ - (Activity type) Renamed ‘Physical activity and exercise’ to ‘Physical activities.’ - (Activity type) Renamed ‘Activity wrap-up and real-life linkage activities’ to ‘Closing-stage activities.’ - (Reclassification of sub-activities) ‘Art activities’ reclassified under ‘Activities mediated by natural elements and arts.’ - (Reclassification of sub-activities) ‘Play with natural objects’ reclassified under ‘Activities mediated by natural elements and arts.’
Sub-activities	- Integrated ‘Discovering my values’ and ‘Self-exploration activities’ into ‘Self-exploration activities.’ - Integrated ‘Noticing emotions’ and ‘Expressing emotions’ into ‘Emotion exploration activities.’ - Integrated ‘Noticing stress’, ‘Expressing stress’, and ‘Finding ways to cope and manage stress ’ into ‘Stress management activities.’ - Integrated ‘Exercise’ and ‘Forest exercise’ into ‘Physical function enhancement exercise.’ - Renamed ‘Noticing thoughts’ to ‘Thought awareness activities.’ - Renamed ‘Noticing the present’ to ‘Present-moment awareness activities.’ - Renamed ‘Finding happiness’ to ‘Happiness exploration activities.’ - Renamed ‘Problem analysis’ to ‘Problem analysis activities.’ - Renamed ‘Enhancing social skills’ to ‘Social skills enhancement activities.’ - Renamed ‘Indoor plant-use activities’ to ‘Indoor plant-care activities’, and ‘Outdoor plant-use activities’ to ‘Outdoor plant-care activities.’ - Renamed ‘Hydrotherapy activities’ to ‘Water-use activities.’ - Renamed ‘Concluding activities’ to ‘Closing activities.’ - Deleted ‘Setting my dreams and goals’, ‘Identifying strengths and weaknesses’, ‘cognitive restructuring and awareness’, ‘Identifying and planning solutions’, ‘Implementing and evaluating solutions’, ‘Tea ceremony’, ‘Play’, ‘Literature activities’, ‘Forest literature activities’, ‘Music activities’, ‘Laughter activities’, ‘Cooking activities’, ‘Storytelling’, and ‘Assigning practice tasks.’
Selection questions for activity types	- (Self-understanding and management activities) Changed ‘activities that develop problem-solving skills’ to ‘activities that help patients manage themselves.’ - (Physical activities) Changed ‘activities that enable the patient to feel physical vitality’ to ‘activities that allow the patient to move their body or enhance physical functions.’ - (Activities mediated by natural elements and arts) Merged into one question: ‘Is there a need for activities in which the patient experiences nature or engages in expressive activities mediated by natural elements and arts?’ due to the integration of activity types.
Precautions	- Changed ‘excessive sensory stimulation’ to ‘excessive sensory intake.’ - Deleted ‘activities that deeply explore the inner self.’

**Table 17:** Activity Types, Selection Questions, Therapy Activities.

Activity Type		Content (Selection Questions and Therapy Activities)	
Purpose-Based Activities	Self-Understanding and Management Activities	Selection Question: *Does the patient need activities that help them understand themselves and others and manage themselves?*	☐Yes ☐No
		• Self-exploration activities • Emotion-exploration activities • Thought-awareness activities • Present-moment awareness activities • Happiness-exploration activities • Problem-analysis activities • Stress-management activities • Social-skills enhancement activities	
Utilization-Based Activities	Introductory-Stage Activities (Essential Components)	No selection question (mandatory program components)	
		• Self-introduction • Ice-breaking • Setting rules	
	Sensory Stimulation and Mind–Body Relaxation Activities	Selection Question: *Does the patient need activities that stimulate the senses and relax the body and mind?*	☐Yes ☐No
		• Awakening the five senses • Stretching • Physical relaxation activities • Breathing techniques • Meditation	
	Physical Activities	Selection Question: *Does the patient need activities that involve physical movement or enhance physical functioning?*	☐Yes ☐No
		• Forest walking • Physical-function enhancement exercise • Yoga	
	Activities Mediated by Natural Elements and Arts	Selection Question: *Does the patient need activities involving nature experiences or expressive activities mediated by natural elements and arts?*	☐Yes ☐No
		• Forest experience • Water-use activities • Play with natural objects • Indoor plant-care activities • Outdoor plant-care activities • Art activities	
	Closing-Stage Activities (Essential Components)	No selection question (mandatory program components)	
		• Closing activities	

**Table 18:** Precautions.

Category	Content	
Physical-condition related Precaution	*The patient may be at risk of allergic reactions during forest activities.*	☐Yes ☐No
	*Certain activities may be unsuitable if the patient has discomfort or a medical condition in a specific body area.*	☐Yes ☐No
	*The patient may feel discomfort if sensory stimulation is excessive or overwhelming.*	☐Yes ☐No
Psychological-condition related Precaution	*The patient feels discomfort with physical contact from others.*	☐Yes ☐No
	*The patient experiences fear or discomfort toward specific individuals (e.g., gender, age group).*	☐Yes ☐No
	*The patient experiences fear regarding situations or activities that evoke certain past events or memories.*	☐Yes ☐No
	*The patient feels burdened when expressing thoughts or emotions in front of others.*	☐Yes ☐No
Location-related Precaution	*The patient feels discomfort engaging in activities conducted in enclosed spaces perceived as confining.*	☐Yes ☐No
	*The patient feels anxiety in certain locations (e.g., near water, dark spaces, narrow spaces, or enclosed areas).*	☐Yes ☐No

## Data Availability

The data presented in this study are available on request from the corresponding author. The data are not publicly available due to privacy.

## References

[1] World Health Organization Depressive Disorder (Depression). https://www.who.int/news-room/fact-sheets/detail/depression.

[2] Organisation for Economic Co-Operation and Development Suicide Rates. https://www.oecd.org/en/data/indicators/suicide-rates.html.

[3] Kim M.-R., Park T.-H., Oh H.-Y. (2023). A study on the development of an empathic chatbot for alleviating depressive symptoms. J. Korea Inst. Inf. Commun. Eng..

[4] National Center for Mental Health Depressive Disorder. https://www.mentalhealth.go.kr/portal/disease/diseaseDetail.do?dissId=36.

[5] Lee M.-S. (2013). Current status and diagnosis of depression in recent years. J. Pharm. Hosp. Assoc..

[6] Gorman J.M. (1996). Comorbid depression and anxiety spectrum disorders. Depress. Anxiety.

[7] National Center for Mental Health (2024). 2024 National Survey of Public Knowledge and Attitudes Toward Mental Health.

[8] Lee H.-S., Kang D.-Y., Korean Society for Clinical Pharmacology (2009). Clinical Neuropsychiatric Pharmacology.

[9] Korea Forest Service Forestry Culture and Recreation Act. https://elaw.klri.re.kr/kor_service/lawView.do?hseq=27993&lang=ENG.

[10] Ibes D., Hirama I., Schuyler C. (2018). Greenspace ecotherapy interventions: The stress-reduction potential of green micro-breaks integrating nature connection and mind–body skills. Ecopsychology.

[11] Kim J. (2021). Effects of a Self-Guided Forest Therapy Program. Master’s Thesis.

[12] Rivieccio R., Meneguzzo F., Margheritini G., Re T., Riccucci U., Zabini F. (2025). Therapist-guided versus self-guided forest immersion: Comparative efficacy on short-term mental health and economic value. Behav. Sci..

[13] Gao D., Shen J., Gao Y., Zhang Z. (2024). The beneficial elements in forest environment based on human health and well-being perspective. Forests.

[14] Li Q., Kobayashi M., Wakayama Y., Inagaki H., Katsumata M., Hirata Y., Hirata K., Shimizu T., Kawada T., Park B.J. (2009). Effect of phytoncide from trees on human natural killer cell function. Int. J. Immunopathol. Pharmacol..

[15] Woo J., Yang H., Yoon M., Gadhe C.G., Pae A.N., Cho S., Lee C.J. (2019). 3-Carene, a phytoncide from pine trees, has a sleep-enhancing effect by targeting the GABAA-benzodiazepine receptors. Exp. Neurobiol..

[16] Kim Y.J., Hong G.L., Kim K.H., Lee H.J., Cho S.P., Joung D., Pack B.J., Jung J.Y. (2023). Effects of phytoncide extracts on antibacterial activity, immune responses, and stress in dogs. J. People Plants Environ..

[17] Donelli D., Meneguzzo F., Antonelli M., Ardissino D., Niccoli G., Gronchi G., Baraldi R., Neri L., Zabini F. (2023). Effects of plant-emitted monoterpenes on anxiety symptoms: A propensity-matched observational cohort study. Int. J. Environ. Res. Public Health.

[18] Dudek T., Marć M. (2025). Terpene content in the air of younger and older forests in southeastern Poland: Implications for forest therapy. Sci. Rep..

[19] Shin W.-S., Oh H.-G. (1996). The Influence of the Forest Program on Depression Level. J. Korean For. Soc..

[20] Sin W.-S., Yeon P.-S., Lee J.-H., Kim S.-G., Joo J.-S. (2007). Effects of forest experiences on anxiety and depression in participants. J. Korean For. Rec. Sci..

[21] Kim W., Woo J., Park S., Lim S. (2012). Effects of forest activities on recovery of patients with depression: A comparative study among forest therapy program group, hospital program group, forest bathing group, and control group. J. Korean For. Sci. Soc..

[22] Yeon P., Kim I., Kang S., Lee N., Kim G., Min G., Chung C., Lee J., Kim J., Shin W. (2022). Effects of urban forest therapy program on depression patients. Int. J. Environ. Res. Public Health.

[23] Yeon P., Kang S., Lee N., Kim I., Min G., Kim G., Kim J., Shin W. (2023). Benefits of urban forest healing program on depression and anxiety symptoms in depressive patients. Healthcare.

[24] Yeon P., Jeon J., Jung M., Min G., Kim G., Han K., Shin M., Jo S., Kim J., Shin W. (2021). Effect of forest therapy on depression and anxiety: A systematic review and meta-analysis. Int. J. Environ. Res. Public Health.

[25] Zhang Z., Ye B. (2022). Forest therapy in Germany, Japan, and China: Proposal, development status, and future prospects. Forests.

[26] Park S., Yeon P. (2025). Analysis of Research Trends in Forest Therapy Using LDA Topic Modeling. J. Korean Inst. For. Recreat..

[27] Korea Forest Healing Forum (2022). A Survey of Nature Prescription Cases Related to Forest Healthcare.

[28] National Institute of Forest Science (2024). Development of Indicators and a Health–Medical Linkage Service Model for Building a Forest Healing Big Data System.

[29] Lee M., Park Y., Eun S.-D., Ho S.H. (2023). Development of a Set of Assessment Tools for Health Professionals to Design a Tailored Rehabilitation Exercise and Sports Program for People with Stroke in South Korea: A Delphi Study. Healthcare.

[30] Son M.-G. (2022). Development of a Diagnostic Assessment Tool for Music Therapy for Children with Attention Deficit Hyperactivity Disorder. Ph.D. Thesis.

[31] Lawshe C.H. (1975). A quantitative approach to content validity. Pers. Psychol..

[32] Park Y.-H., Jang J.-H. (2019). A Delphi Study to Develop Performance Evaluation Indicators for Occupational Safety and Health Education Projects. HRD Res..

[33] Ayre C., Scally A.J. (2014). Critical values for Lawshe’s content validity ratio: Revisiting the original methods of calculation. Meas. Eval. Couns. Dev..

[34] Lee E., Yoo R., Park C., Kim J. Analysis of activity components in domestic forest therapy programs. Proceedings of the Korean Forest Recreation Society Conference.

[35] Kim Y.H., Jeong D.W., Park B.J. (2019). Classification of domestic forest therapy programs. J. Korean Inst. For. Recreat..

[36] Jeon J.-Y., Kang S.-N., Kim J.-G., Yeon P.-S. (2022). Analysis of types of domestic forest therapy programs for improving depression. J. Korean For. Rec. Sci..

[37] Watkins E.R. (2008). Constructive and unconstructive repetitive thought. Psychol. Bull..

[38] Nolen-Hoeksema S. (2000). The role of rumination in depressive disorders and mixed anxiety/depressive symptoms. J. Abnorm. Psychol..

[39] Kim G., Kang S., Paek K., Lee N., Min G., Seo Y., Park S., Park S., Choi H., Choi S. (2025). Analysis of program activities to develop forest therapy programs for improving mental health: Focusing on cases in Republic of Korea. Healthcare.

